# Management and Outcomes of Bilateral Acetabular Fractures: A Critical Review of the Literature

**DOI:** 10.1007/s43465-021-00593-1

**Published:** 2022-01-08

**Authors:** Vasileios K. Mousafeiris, Anastasia Vasilopoulou, George D. Chloros, Michalis Panteli, Peter V. Giannoudis

**Affiliations:** 1 Orthopedic Surgery Working Group, Society for Junior Doctors, Athens, Greece; 2 St. Andrews General Hospital of Patras, Patras, Greece; 3 Korgialeneio Mpenakeio Hellenic Red Cross Hospital, Athens, Greece; 4 Academic Department of Trauma and Orthopaedics, School of Medicine, University of Leeds, Leeds General Infirmary, Clarendon Wing, Floor D, Great George Street, Leeds, LS1 3EX UK; 5 NIHR Leeds Biomedical Research Center, Chapel Allerton Hospital, Leeds, UK

**Keywords:** Bilateral acetabular fractures, Trauma, Seizures

## Abstract

**Background:**

Bilateral acetabular fractures constitute a rare entity, and their optimal management is unknown.

**Materials and Methods:**

A systematic literature search was conducted in PubMed, Embase and Cochrane Library between 1995 and 2020. Inclusion criteria were studies presenting cases of bilateral acetabular fractures and reporting outcomes. Extracted data included patient demographics, injury mechanism, fracture classification, associated injuries, management and outcomes.

**Results:**

Thirty-seven studies (47 cases; 35 males vs 12 females) were included. Mean age was 46 years old (range 13–84) and mean follow-up was 19.8 months (range 1.5–56). High-energy injuries (49%) and seizures (45%) were the most common injury mechanisms. Fracture type distribution differed according to injury mechanism. Treatment was surgical in 70% of cases (75% open reduction and internal fixation vs 25% acute total hip arthroplasty). Outcomes were excellent/good in 58% of patients. Complications included heterotopic ossification (11%), nerve injury (11%), degenerative arthritis (6%), DVT (6%), and infection (3%).

**Conclusions:**

Bilateral acetabular fractures most commonly occur either after trauma or seizures and are commonly managed operatively. They are not devoid of complications, however, more than half (58%) achieve complete functional recovery.

## Introduction

Acetabular fractures represent complex injuries, with an incidence of just 3 per 100,000 population/year [1]. Most common causes include road traffic accidents (RTA), followed by falls from height and pedestrians accidents [1]. Because of the high-energy mechanism of injury (MOI), associated injuries are common and are, therefore, related with increased morbidity and mortality [1, 2].

Bilateral acetabular fractures on the other hand have an extremely rare occurrence with limited reports in the literature. Little is known about their demographics, MOI, fracture configuration, treatment approach, outcomes, and complications. To our knowledge, there have been no comprehensive reviews of this entity.

The purpose of this study is to summarize the available evidence on bilateral acetabular fractures, including management and outcomes.

## Patients and Methods

A systematic search of the literature was conducted to identify the available evidence about bilateral acetabular fractures. All studies identified in the English and German published from Jan 1995 to Dec 2020 via the Cochrane Library, Embase via OVID and MEDLINE (through PubMed) electronic databases were assessed. Specific search strings were formulated for each database using the following keywords and/or MeSH terms: (1) ("bilateral"[All Fields] OR "bilaterally"[All Fields] OR "bilaterals"[All Fields]) AND (2) "acetabul*"[All Fields] AND "fractur*"[All Fields] or Bilateral acetabul* fractur*. This study was conducted according to the 2009 Preferred Reporting Items for Systematic Review and Meta-analysis (PRISMA) statement [3].

Inclusion criteria were studies reporting on cases of bilateral acetabular fracture management, outcomes and complications. Exclusion criteria were unilateral acetabular fractures, biomechanical studies, animal studies, review articles, post-mortem studies, foreign language literature, editorials, comments, opinions, letters to the Editor, published abstracts and errata (unless they provide original data). The reference lists of the eligible studies and relevant review articles were cross-checked to identify additional relevant studies.

### Data Extraction

Data extracted from the eligible studies included patient demographics (age, sex, comorbidities), MOI, fracture classification, associated injuries (orthopaedic and non-orthopaedic), procedures performed, classification, outcomes and complications. All data was inserted in an electronic database for subsequent analysis.

## Results

Of the 254 studies screened by title/abstract, 40 papers [4–43] were identified and full text was checked for eligibility. Three case reports [4, 42, 43] were further excluded at this stage, as they did not report relevant outcomes. Thirty-six case reports [5–33, 35–41] and 1 short case series [34] were finally included and formed the basis of this review (Table 1).

**Table 1:**
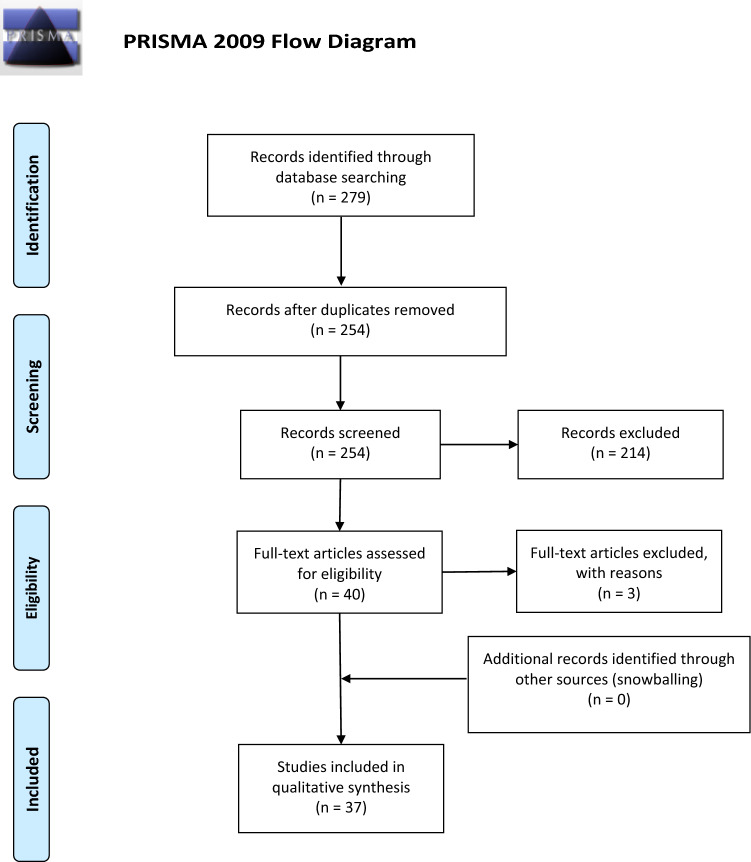
PRISMA flow diagram

### Patient Demographics and MOI (Table 2)

**Table 2:** Patient demographics and mechanism of injury

Mechanism of injury	% (number of cases)	Male/female ratio	Mean age in years (range)
1. High-energy injuries			
Total	49% (23)	3.6/1	38 (15–82)
RTA	70% (16)		
Falls	13% (3)		
Direct blow	9% (2)		
Crush injury	4% (1)		
Not reported	4% (1)		
2. Seizures			
Total	45% (21)	3.2/1	55 (13–84)
3. Insufficiency fractures			
Total	6% (3)	½	47 (23–70)

Forty-seven patients (35 males, 12 females) with a mean age of 46 years (range 13–84 years) were analysed. Follow-up was reported in 72% (34/47) of the cases with a range of 1.5–56 months, (mean 19.8 months) [5–16, 18–28, 30, 32–41]. The most common MOI was high energy in 49% (23/47; male/female ratio (M/F) = 3.6/1) [8, 12, 13, 15, 20, 25, 26, 29, 30, 33, 34, 36, 38, 39], followed by seizures in 45% (21/47; M/F = 3.2/1) [6, 9–11, 14, 16, 17, 19, 21–24, 27, 31, 32, 35, 37, 40, 41], and lastly by insufficiency fractures secondary to osteoporosis 6% (3/47) (Table 2) [5, 7, 28]. High-energy injuries were secondary to RTAs (70%; 16 patients) [12, 13, 15, 20, 26, 29, 30, 33, 34], falls from height (13%; 3 patients) [8, 38, 39], direct blows (9%; 2 patients) [25, 30], crush injury (4%; 1 patient) [34], and unknown mechanism (4%; 1 patient) [18]. Mean age was 38 years (range 15–82) for the high-energy group [8, 12, 13, 15, 18, 20, 25, 26, 29, 30, 33, 34, 36, 38, 39], 55 years (range 13–84) for the seizure group [6, 9–11, 14, 16, 17, 19, 21–24, 27, 31, 32, 35, 37, 40, 41] and 47 years (range 23–70) for the insufficiency fractures group [5, 7, 28].

### Fracture Classification (Table 3)

**Table 3:** Fracture classification (Letournel and Judet [44]) per mechanism of injury by A fracture type distribution, B most common fracture type and C most common combination of injuries

Group by mechanism of injury	A. Fracture type distribution (left vs right)											Total	B. Most common fracture type on either side	C. Most common bilateral combination of injury
	Side	PW	PC	AW	AC	TV	ABC	TPW	T	ACPH	PWC			
1. High energy	R	5	0	4	2	2	1	5	0	2	1	22	1. PW 11/45 (24%) [12, 18, 20, 34, 38] 2. TPW 11/45 (24%) [13, 20, 34, 38] 3. AW 6/45 (13%) [25, 26, 34] 4. AC 6/45 (13%) [13, 15, 29, 39]	1. TPW 4/22 (18%) [20, 34, 38] 2. AW + PW 3/22 (14%) [13, 34]
	L	6	0	2	4	3	1	6	0	0	1	23		
	Total	11	0	6	6	5	2	11	0	2	2	45		
2. Seizures	R	1	0	3	4	0	5	1	1	1	0	16	1. ABC 12/30 (40%) [6, 21, 31, 32, 37, 40, 41] 2. AC 7/30 (23%) [10, 14, 27, 37] 3. AW 4/30 (13%) [6, 32, 37]	ABC 4/12 (33%) [6, 21, 40, 41]
	L	0	0	1	3	0	7	0	1	1	1	14		
	Total	1	0	4	7	0	12	1	2	2	1	30		
3. Insufficiency fractures	R	0	0	0	0	0	0	0	0	1	0	1	ACPH 1/1 (100%) [7]	ACPH 1/1 (100%) [7]
	L	0	0	0	0	0	0	0	0	1	0	1		
	Total	0	0	0	0	0	0	0	0	2	0	2		
Overall	r	6	0	7	6	2	6	6	1	4	1	39	1. ABC 14/77 (18%) 2. AC 13/77 (17%) 3. PW 12/77 (16%) 4. TPW 12/77 (16%)	TPW 4/35 (11%) [20, 34, 38] ABC 4/35 (11%) [6, 21, 40, 41] AW + PW 3/35 (9%) [13, 34]
	L	6	0	3	7	3	8	6	1	2	2	38		
	Total	12	0	10	13	5	14	12	2	6	3	77		

*PW* posterior wall, *PC* posterior column, *AW* anterior wall, *AC* anterior column, *TV* transverse, *ABC* associated both column, *TPW* transverse with posterior wall, *T* T-shaped, *ACPH* anterior column (or wall) posterior hemitransverse, *PCW* posterior column and posterior wall, *R* right, *L* left

The Letournel and Judet classification [44] was reported in 77% (36/47) of cases [6, 7, 10, 12–15, 17, 18, 20, 21, 25–27, 29–41], whereas the remaining fractures were not classified [5, 8, 11, 16, 19, 22, 24–28]. Overall, the most frequent type was associated both column fracture (ABC) with 18%, followed by anterior column (AC) with 17%, while the least common was posterior wall and column (PWC) with 4% and T-shaped (*T*) with 3%.

For the high-energy injuries, the most common fracture types on either side were PW [12, 18, 20, 34, 38] and TPW [13, 20, 34, 38] with 24% each, whereas, the most common fracture combination was bilateral TPW (18%) [20, 34, 38], followed by AW on one side and PW on the contralateral side (14%) [13, 34]. In the seizure group, the most common fracture types on either side were ABC (40%) [6, 21, 31, 32, 37, 40, 41], followed by AC (23%) [10, 14, 27, 37]. The most common fracture combination was bilateral ABC in 31% of cases [6, 21, 40, 41], whereas other combinations were inconsistent, in the sense that in the remaining cases, the fracture on the right is different from the fracture on the left [9–11, 14, 16, 17, 19, 22–24, 27, 31, 32, 35, 37]. For the Insufficiency fractures group, only 1 fracture combination is reported: AC and posterior hemitransverse [7].

### Associated Injuries

Those were reported in 89% of the cases (42/47 cases) [5–14, 16, 17, 19–24, 26–34, 36–41]. The most common associated injuries were upper limb injuries (26%) [8, 10, 13, 17, 19, 31, 32, 34, 37, 39], pelvic fractures (23%) [7, 11, 12, 34, 36, 38, 39, 41], femoral fractures (19%) [10, 16, 31, 32, 34, 38], spine injuries (14%) [10, 27, 30, 31, 34], acetabular fracture associated with hip dislocation (23%) [10, 14, 20, 22, 23, 26, 33, 38, 40], chest injuries (12%) [13, 29, 34, 36], lower leg fractures (7%) [30, 34, 36], and head injury (2%) [26].

### Treatment

Operative treatment versus non-operative treatment was undertaken in 70% [5–8, 10, 12–18, 20, 21, 23, 27, 28, 31, 33, 34, 36–40] and 30% [9, 11, 18, 19, 22, 25, 26, 29, 30, 32, 35, 41] of cases, respectively.

#### Operative Management

Operative management of bilateral acetabular fractures has been reported as a single-stage, a 2-stage or even a more than 2-stage procedure.

Overall, single-stage fixation was reported in 52% of cases [7, 8, 13, 16, 17, 20, 23, 27, 34, 36, 37, 39], a 2-stage in 45% of cases [5, 10, 12–15, 21, 26, 28–30, 33, 34, 38, 40], whereas in 1 case (3%) a 4-staged operative management [6] was reported (Table 4). The mean time elapsed between stages ranged from 1 day to 3 months [5, 10, 12–15, 21, 26, 28–30, 33, 34, 38, 40].

**Table 4:** Staging in operatively treated bilateral acetabular fractures per MOI group

MOI group	Single stage	2-stages	> 2 stages
High energy	10 (56%)	8 (44%)	0 (0%)
Seizures	5 (50%)	4 (40%)	1 (10%)
Insufficiency fractures	1 (33%)	2 (67%)	0 (0%)
OVERALL	16 (52%)	14 (45%)	1 (3%)

In the high-energy group, 17% (4/23) of cases were treated conservatively [25, 26, 29, 30].Of the 83% (19/23) of cases that were treated operatively [8, 12, 13, 15, 18, 20, 33, 34, 36, 38, 39], 90% (18/19) give further details on stages of fixation [8, 12, 13, 15, 20, 25, 26, 29, 30, 33, 34, 36, 38, 39]. Fifty-six percent of cases (10/18) were treated in a single stage [8, 13, 20, 34, 36, 39], while a 2-stage procedure was undertaken in 8/18 cases (44%) [12, 15, 33, 34, 38].

In the seizure group, 48% (10/21) of cases were treated conservatively [9, 11, 19, 22, 24, 32, 35, 41]. Of the 52% (11/21) of cases that were treated operatively 90% (10/11) give further details on stages of fixation [6, 9–11, 14, 16, 17, 19, 21–24, 27, 31, 32, 35, 37, 40, 41]. Single-staged procedure was performed in 50% (5/10) [16, 17, 23, 27, 37], 2- staged procedure was performed in 40% (4/10) [10, 14, 21, 40] and 4-staged procedure was carried out in 10% (1/10) [6].

In the insufficiency fractures group 33% (1/3) cases were treated via single-stage procedure [7], while 67% (2/3) cases were treated via 2-staged procedure (5, 28).

#### Type of Surgery and Surgical Approach Used (Tables 5 and 6)

**Table 5:** Treatment modality per MOI group per fracture

Side	Right			Left			Combined		
Treatment	ORIF	THA	Cons	ORIF	THA	Cons	ORIF	THA	Cons
High energy	16	0	4	14	0	6	30 (75%)	0 (0%)	10 (25%)
Seizures	7	5	14	6	6	14	13 (25%)	11 (21%)	28 (54%)
Insufficiency fractures	1	2	1	1	2	1	2 (25%)	4 (50%)	2 (25%)
Overall	24	7	19	21	8	21	45 (45%)	15 (15%)	40 (40%)

*ORIF* open reduction and internal fixation, *THA* total hip arthroplasty, *Cons* conservative

**Table 6:** Overall prevalence of surgical approaches used per MOI group and fracture

Groups	Anterior approaches			Posterior approaches			Combined approach	Overall
	Modified Stoppa/AIP	Ilioinguinal	Iliofemoral	Kocher–Langenbeck	Iselin	Moore		
High energy	5 (18%)	3 (11%)	1 (4%)	16 (57%)	0	3 (11%)	0	28 (70%)
Seizures	2 (20%)	2 (20%)	0	2 (20%)	2 (20%)	0	2 (20%)	10 (25%)
Insufficiency fractures	2	0	0	0	0	0	0	2 (5%)
Total	9 (23%)	5 (13%)	1 (3%)	18 (45%)	2 (5%)	3 (8%)	2 (5%)	40 (100%)

Anterior approach: Ilioinguinal, iliofemoral or modified Stoppa approach. Posterior approach: Kocher–Langenbeck, Iselin or Moore. Combined approach refers to both anterior and posterior approaches done during the same stage

The majority of the fractures in the high-energy group (75%) were treated with open reduction and internal fixation (ORIF) [8, 12, 13, 15, 18, 20, 33, 34, 36, 38, 39], with the remaining 25% being treated conservatively [25, 26, 29, 30]. No total hip arthroplasty (THA) was performed in the high-energy group (Table 5) [8, 12, 13, 15, 18, 20, 25, 26, 29, 30, 33, 34, 36, 38, 39].

In the seizure group, the majority of fractures was treated conservatively (54%), with 25% undergoing ORIF [6, 10, 17, 21, 23, 27, 31] and 21% treated with THA [6, 14, 16, 37, 40]. In this group, 4 cases were treated initially conservatively and then received late THA bilaterally (Table 5) [6, 14, 37, 40].

In the insufficiency fractures group 50% of the fractures underwent THA [5, 28], 25% were treated conservatively, and 25% underwent ORIF [7]. One case originally managed conservatively, underwent THA at a later stage (Table 5) [5].

Overall, five cases were treated initially conservatively, but then received late THA bilaterally [5, 6, 14, 37, 40]. There were, also, three cases where the two sides were treated with a different procedure [10, 34]. More specifically, in the study of Gill et al. [10] the patient underwent ORIF on the right side and THA on the left side, due to an intracapsular neck femur fracture. In two cases in the study of Steven et al. [34], the patients underwent ORIF on the right side and were treated conservatively on the left side.

In the high-energy group, 68% of the fractures underwent a posterior approach [13, 18, 33, 34, 38], while 32% were approached anteriorly [8, 12, 15, 34]. In the seizure group 40% of the fractures underwent anterior approach [23, 27], followed by 40% that underwent posterior [6, 21] and 20% that underwent combined approach [21]. In the insufficiency group, all fractures underwent anterior approach [7] and the approach was not reported in 3 cases treated with ORIF (Table 6) [10, 17, 31].

#### Length of Surgery, Need for Blood Transfusion and ICU Stay

Length of surgery was not reported throughout the studies. The need for blood transfusion is reported in only five patients [11, 13, 24, 35] and a mean of four units of packed red blood cells (range 3–6 units) is reported in two of those patients [24, 35]. Post-operative ICU requirement was reported in only eight patients [10, 11, 13, 23, 24, 29, 41]; the mean length of stay was 3.2 days [1–7 range], while in three cases [10, 13, 41] length of ICU stay is not reported.

#### Rehabilitation

Rehabilitation protocol was reported in 15 cases [6, 9, 11–13, 15, 21–23, 26, 32, 33, 35, 37, 39]. 9 cases [6, 9, 11, 21–23, 32, 35, 37] in the seizure group and 6 cases[12, 13, 15, 26, 33, 39] in the high-energy group, while in the remaining cases, no post-operative physiotherapy and weightbearing protocols are reported [5, 7, 8, 10, 14, 16–20, 24, 25, 27–31, 34, 36, 38, 40, 41]. In those reported, no patient was allowed to immediate fully weight bear. Patients were advised to avoid weight-bearing for at least 6 weeks in the majority of studies [12, 15, 21, 23, 33, 35].

### Outcomes

Outcomes were reported descriptively, i.e. excellent/good vs fair vs poor in 85% (40/47) of cases [5–18, 20–26, 28, 30–40], whereas in the rest, no outcomes were reported [19, 27, 29, 34, 41].

Overall, there were 58% excellent/good outcomes with complete functional recovery and independent mobilization [5, 7, 9, 10, 13, 15–18, 22, 25, 26, 28, 31, 33–35, 38–40], 22% fair with limited range of motion of both hips and moderate pain [6, 8, 23, 30, 32, 34, 36, 37], and 20% poor outcomes with limited activity and walking ability (Table 7) [11, 12, 14, 20, 21, 24, 34].

**Table 7:** Outcomes according to MOI

Groups	Descriptive outcomes—40 cases		
	Excellent/good	Fair	Poor
High energy	12 [13, 15, 18, 25, 26, 33, 34, 38, 39] (60%)	5 [8, 30, 34, 36] (25%)	3 [12, 20, 34] (15%)
Seizures	8 [9, 10, 16, 17, 22, 31, 35, 40] (47%)	4 [6, 23, 32, 37] (24%)	5 [11, 14, 21, 24] (29%)
Insufficiency fractures	3 [5, 7, 28] (100%)	0	0
Overall	23 (58%)	9 (22%)	8 (20%)

	Functional scores—14 cases		
	Harris hip Score	Oxford Hip Score	Merle D’Aubigne and Postel Criteria
High-energy	2 [13]	6 [34]	1 [18]
Seizures	3 [16, 21, 37]	0	1 [22]
Insufficiency fractures	1 [28]	0	0
Overall	6	6	2

According to injury group, excellent/good outcomes were reported in 60% of the high-energy patients [13, 15, 18, 25, 26, 33, 34, 38, 39], 47% of the seizures patients [9, 10, 16, 17, 22, 31, 35, 40], and in 100% of the insufficiency fracture group [5, 7, 28].

Functional scores were reported in 30% (14/47) of cases [13, 16, 18, 21, 22, 28, 34, 37]. The mean Harris hip score reported in 43% (6/14) of cases was 85 (good) [13, 16, 21, 28, 37] and the Oxford Hip Score reported in 43% (6/14) of cases was 20–29 in 1 case (moderate to severe arthritis), 30–39 in 2 cases (mild to moderate arthritis) and 40–48 in 3 cases (satisfactory joint function) [34]. The Merle D'Aubigne and Postel Criteria were reported in 14% (2/14) cases as good [18, 22].

We examined whether the presence of associated pelvic injury has any impact on the outcome of patients with bilateral acetabular fractures. Eight papers report on associated pelvic injuries [7, 11, 12, 34, 36, 38, 39, 41]. However, no association was noted as pelvic injury did not affect outcome.

### Complications

Complications were only reported in 36/47 cases (77%) [5–8, 12–17, 20–26, 28, 30, 32–40]. The most common complication is heterotopic ossification 4/36 (11%) [6, 21, 33, 37] and nerve injury 4/36 (11%) [12, 20, 33, 36], followed by degenerative arthritis 2/36 (6%) [6, 30], DVT 2/36 (6%) [14, 36], infection 1/36 (3%) [8] and abductor muscle weakness 1/36 (3%) [24] (Table 8). Finally, mortality was only reported in two cases and was unrelated to surgery [41].

**Table 8:** Complications reported in 75% (36/47) of cases

Complications	Frequency % (nr. of cases)
Heterotopic ossification [6, 21, 33, 37]	11% (4)
Nerve injury [12, 20, 33, 36]	11% (4)
Degenerative arthritis [6, 30]	6% (2)
DVT [14, 36]	6% (2)
Infection [8]	3% (2)
Abductor muscle weakness [24]	3% (1)

## Discussion

Acetabular fractures represent complex injuries, usually associated with high-energy trauma and remain a challenge to manage [1, 2, 45, 46]. The literature is scarce regarding bilateral injuries, with only 47 cases from 37 studies being identified. The results show a male preponderance of 75%, which is similar to the unilateral injuries [45–47]; this is comparable in both high-energy and seizure groups (78% and 76%). As in the case of unilateral injuries, a big proportion of bilateral injuries are the result of a high-energy mechanism (49%) in the younger population with a mean age of 38 years. However, this study also identified that a significant proportion (45%) of these injuries occurs secondary to seizures, mainly involving an older population with a mean age of 55 years old. Finally, only 3 cases of bilateral insufficiency fractures have been reported (Table 2) [5, 7, 28]. Interestingly, there was a 23-year-old female patient with pregnancy-induced osteoporosis in the latter group [5]. The reported mean age in the unilateral literature is closer to the high-energy group, a finding which is not surprising as they share a common mechanism [45–48]. The Letournel and Judet classification system [44] remains the most commonly used classification system for acetabular fractures [48, 49], utilised by all included papers that report on classification [6, 7, 10, 12–15, 17, 18, 20, 21, 25–27, 29–41].

The most common fracture types in our study were ABC, AC, TPW and PW with frequencies similar to those reported in the literature [1, 45, 46, 50–52]. PWC and T-shaped were the least common, whilst no cases with PC fractures we reported. In the high-energy group, the most common fracture types were similar to those reported in the literature (PW and TPW 24% each, 48% combined) [45, 46]. In the seizure group, the most common fracture types included the anterior wall and column (AW, AC and ABC fractures; 13%, 23% and 40% respectively, 75% combined). Although AC fractures are not as frequent in the unilateral population, their frequency has been reported to be higher in the elderly population [53], which may explain their higher incidence in the seizure group. Furthermore, contrary to the literature, AW fracture in our study was reported almost eight times more compared to the acetabular fractures in general [46, 52]. Even comparing with the elderly population (aged > 60), this still remains about four times more [53]. Lastly, in the insufficiency fractures group, ACPH was the only type reported, in agreement with Papadakos et al. [54], where ACPH was the most common type in acetabular fractures attributed to low energy mechanisms. As far as the different combinations of the bilateral injuries, the high-energy group had TPW and AW + PW, in 18% and 14% of cases, respectively. In contrast, the seizure group showed an ABC pattern in the majority of cases (33%), see Table 4. Given the simultaneous continuous bilateral contraction of the strong pelvi-trochanteric musculature during a seizure episode [35, 49, 55], which forces the femoral head medially [56], the authors hypothesized that, in the seizure group, the fractures are possibly added every few seconds of the seizure episode in a cumulative way.

More than 40% of acetabular fractures have associated injuries, with the most common being lower extremity (21.8%), followed by upper extremity (9.6%), and pelvis/spine in 5.5%, whereas multiple fractures are reported in 22.8% of cases [46]. In this study, the majority of associated injuries were upper extremity (26%), followed by pelvic (23%), femur (19%) and spinal (14%) fractures. Of note, while in the high-energy group similar frequencies are shown compared to the general literature, in the seizure group, the incidence of associated upper extremity, femoral fractures and spine injuries was 64%, 62% and 50%, respectively. This is commensurate with a recent meta-analysis which showed an increased general fracture risk in patients with epilepsy compared to the general population, particularly with hip, forearm and spine fractures [57]. Finally, chest injuries showed a similar prevalence, whereas head injuries were underreported in the literature [46]. The authors tried to assess whether the treatment approach changes with the nature and presence of associated injuries. However, no pattern was identified regarding differentiation of the treatment approach based on the presence or absence of pelvic or hip injuries. For example, all four cases with sacral fractures, as well as all three cases that had pelvic fractures were treated differently and in the case of hip fractures, almost half received ORIF and half received THAs.

Treatment options varied and included conservative vs surgical (ORIF or THA with either single- or multi-staged operations for both sides). Overall, 45% underwent ORIF, with 40% treated either initially or ultimately conservatively, while the rest (15%) underwent THA. Interestingly, almost half of the cases in both the high energy and the seizure group were treated with single-stage procedures and the other half with two-staged procedures, revealing no difference in treatment choice regarding the injury mechanism and the different fracture patterns involved (single-staged vs 2-staged; 56% vs 44% in high-energy group, 50% vs 40% in seizure group and 52% vs 45% overall). ORIF remains the “gold standard” treatment for both unilateral and bilateral acetabular fractures [45, 46, 49]. The Kocher–Langenbeck was the most commonly used approach (45%) in our study, in agreement with the literature (48.7–54.8%) [46, 52]. Moreover, the Stoppa approach was utilized in almost a quarter of our patients (23%), almost 6 times more frequently than in the unilateral literature [46, 52]. Although, the Ilioinguinal approach is reported as the second most common approach (21.9–25.9%) [46, 52], in our study it was performed much less frequently (13%). The iliofemoral approach and combined approaches were performed slightly less frequently (Iliofemoral: 3% vs 1.1–12.4% and combined: 5% vs 9.2–10.1) [46, 52]. Of note, 70% of the ORIF procedures were performed in the high-energy group, almost three times more than those reported in the seizure group (25%).

THA was the treatment of choice in 15% of the total fractures of our study but was only performed in the seizure group. More specifically, four cases from the seizure group were treated initially conservatively, and then underwent a late THA. This may be explained by the fact that the high-energy trauma cases are higher impact injuries and require more urgent treatment with ORIF, whereas in seizure group the injury is lower impact, so more patients are treated initially conservatively and receive elective THAs afterwards. In line with that, Papadakos et al. reported that 21.1% of the low energy mechanism acetabular fractures were treated initially conservatively and subsequently received late THA [54]. In unilateral acetabular fractures in the elderly, especially when femoral head fractures are present, early stage THA has shown good clinical results when combined with stable fracture fixation [58, 59]. In addition, THA is a treatment option for secondary arthritis due to non-union, malunion of previously conservatively managed or operated acetabular fractures.

Non-operative treatment is usually reserved among others, for minimally displaced fractures, for patients with significant comorbidities and for the elderly with severe osteoporosis [48, 56, 60]. In this study, almost half of the patients in the seizure group were treated conservatively (48%), while another 19% were treated initially conservatively and received late THAs. In contrast, only 17% of the high-energy group patients were treated conservatively and in another 9%, one side was treated operatively and the other non-operatively. In line with that, Papadakos et al. [54] reported that in low energy mechanism acetabular fractures, 39.4% were treated conservatively and another 21.1% were treated initially conservatively and then received THA, while only 31% underwent ORIF. It is not known why more patients in the seizure group were treated conservatively, but it can be assumed that the risk for ORIF hardware failure or THA dislocation during a subsequent seizure episode and the poor bone quality both as side effect of the antiepileptic medications and due to their increased age, as well as increased medical comorbidities and perioperative morbidity led the surgeons to stall operative treatment. Nevertheless, the choice of treatment approach is frequently directed by the fracture pattern, so a change in the fracture proportion would mandate a change in the approach used.

As far as rehabilitation, this has been reported in 15 cases [6, 9, 11–13, 15, 21–23, 26, 32, 33, 35, 37, 39], including 9 cases in the seizure group [6, 9, 11, 21–23, 32, 35, 37] and 6 in the high-energy group [12, 13, 15, 26, 33, 39]. The majority of patients were advised to avoid full weight-bearing for at least 6 weeks [12, 15, 21, 23, 33, 35].

Excellent/good functional outcomes are reported in 58% of the cases, which is significantly lower than in the unilateral literature (73–80%) [46, 47, 52, 60, 61]. Although, due to short-term follow-up period (< 2 years) further improvement or deterioration of the outcomes cannot be excluded, Giannoudis et al. [52] reported that studies with longer follow-up had better functional outcomes. In this study, fair and poor outcomes are reported in 23% and 20% respectively, compared to the unilateral literature (20% vs 27%) [46, 47, 52, 60, 61].

The most common complications reported included traumatic nerve palsy in 11% of cases, and heterotopic ossification in another 11%. These occurred less frequently than in unilateral acetabular fractures [16% [52] and 19–25.6% [46, 52]], respectively. In addition, post-traumatic osteoarthritis had an incidence of 6% in this series compared to 17.6–19.8% reported in the literature [46, 52], which may be attributed to the short-term (< 2 year) follow-up in the reported studies. Remaining complications including DVT (6%), local infection (3%) and abductor muscle weakness (3%) are reported, which are comparable to the reported literature in unilateral injuries (DVT: 4.3–5.2%, local infection: 4.4–4.5%) [46, 52].

This study has limitations. The majority of the included studies are case reports and therefore of low quality. Another limitation is that no study reports on ISS (injury severity score), which is an important factor in deciding type and timing of treatment. The exact mode of treatment is also not uniform in the included studies. Interestingly, 16% of the cases do not report whether the operations were performed in a single or multiple stages. Regarding combined pelvic and acetabular fractures or other associated orthopaedic injuries there are no protocols established defining early and definitive fixation strategy. In addition, length of surgery and intra-operative blood loss is not reported in any study. The need for blood transfusion is reported in only five patients. Rehabilitation protocol is reported in 15 cases with no clear functional protocols.

## Conclusion

Bilateral acetabular fractures are complex injuries which remain a challenge to manage. They are, mostly, caused by high-energy injuries or seizures. Fracture types of the high-energy group are similar to the unilateral types reported in the literature, while those of the seizure group similar to the types reported mostly in the elderly. ORIF remains the standard method of treatment of the high-energy group, similar to the unilateral fractures, while in the seizure group, bilateral fractures are treated either conservatively, with ORIF, or THA. Larger series that address the aforementioned limitations of the literature are needed to provide further insight and guidelines for orthopaedic trauma surgeons managing bilateral acetabular fractures.
